# Jiangtang Tongmai Prescription Reduced Diabetic Lung Injury Through SnoN and TGF-β1/Smads Signaling Pathway

**DOI:** 10.3389/fendo.2022.846583

**Published:** 2022-06-17

**Authors:** Nian Ding, Chenghong Zheng

**Affiliations:** ^1^ Clinical College of Traditional Chinese Medicine, Hubei University of Chinese Medicine, Wuhan, China; ^2^ Medical Ward, Wuhan Hospital of Traditional Chinese Medicine, Wuhan, China

**Keywords:** JTTMP, TGF-β1/Smads signal, SnoN protein, diabetic lung injury, diabetes

## Abstract

By establishing a rat diabetes model in rats with intervening treatment by Jiangtang Tongmai Prescription (JTTMP), this study explored the restorative pairing effect of JTTMP on diabetic lung injury. The model of type II diabetes model was used to establish the rat diabetes model, using a high-fat diet and streptozotocin (STZ) induction. Different doses of JTTMP and metformin were administered as a therapeutic to intervene, and blood was collected to assess the blood glucose level of each group of rats. HE (Hematoxylin and eosin (H&E) staining was performed to detect the morphological changes in rat lung tissue and enzyme-linked immunoassay ELISA was used to detect and quantify the expression of interleukin (IL)-6, TNF tumor necrosis factor-ɑa, and IL-1β in serum and the lung tissue of each group of rats. The level expression of TGF-β1 [transforming growth factor (TGF)-β1), SnoN (transcriptional co-repressor Ski-N terminal (SnoN)], Smad2, Smad3, Smad7, and other signaling pathway proteins were assessed by Western blot. In comparison with the normal control (NC) group, rats in the diabetes model (DM) group lost weight and showed significantly increased blood sugar levels. The levels of TGF-β1 and Smad2/3 were increased in the DM group but Smad7 decreased. After 8 weeks of JTTMP intervention, the level of TGF-β1 and Smad2/3 decreased but Smad7 increased, blood sugar decreased significantly and the expression of inflammatory factors in lung tissue decreased. Therefore, JTTMP may activate SnoN and the downstream TGF-β1/Smads signaling pathway to repair diabetic lung injury, which suggests its application has potential for future clinical treatment of diabetes with lung injury.

## Introduction

Diabetes mellitus is a metabolic disease that presents with chronically increased glucose levels. Diabetes is clinically characterized by hyperglycemia, with symptoms such as polydipsia, polyuria, polyphagia, and weight loss. In addition to high blood sugar, it is often accompanied by a series of metabolic disorders related to protein, fat, water, and electrolyte processing. Without timely and effective treatment it can cause disease to multiple systems of the body, including diabetic retinopathy, nephropathy, diabetic foot, diabetic nerve disease, and more severe cases such as diabetic ketoacidosis, diabetic lactic acidosis, and diabetic nonketotic hyperosmolar coma (1). Recently, studies have found that the lungs of diabetic patients also show fibrous tissue-like changes similar to the myocardium and kidney, which indicates that pulmonary fibrosis is one of the complications of diabetes (2, 3). The Jiangtang Tongmai Prescription (JTTMP) treatment can be used to treat diabetes as it effectively reduces blood sugar (4, 5), but whether it plays a role in the treatment of diabetic lung injury still needs to be explored. Considering this, we used streptozotocin (STZ) to establish a rat model of diabetes in which to observe the degree of lung injury (6, 7). This was then followed by an intervention with JTTMP to study the effect on diabetic lung injury and its targets and explore the mechanism of JTTMP against lung injury in the treatment of diabetes.

## Materials and Methods

### Materials

The JTTMP Traditional Chinese medicine and STZ were freshly prepared in our laboratory. Metformin (M107827, Aladdin); blood glucose test strips for rat interleukin (IL)-6, tumor necrosis factor (TNF)-ɑ, IL-1β factor enzyme-linked immunoassay (ELISA) detection kit (RA20607, RA20035, RA20020, Bioswamp); eosin, hematoxylin, neutral resin (E8090, G1140, G8590, Solarbio); Protein Marker, BCA protein concentration determination kit (XY-MY-0112, XY-MY-0096, Shanghai Xuanya); polyvinylidene fluoride membrane, enhanced chemiluminescence reagent (XF-P3360, ZDSJ140, Xinfan company); Tween-20 (PW0028, LEAGEN company); RIPA tissue cell rapid lysate (BL504A, biosharp); transforming growth factor (TGF)-β1, Smad2, Smad3, Smad7, p-Smad7, Ski-N terminal (SnoN), glyceraldehyde 3-phosphate dehydrogenase (GAPDH) primary antibody (PAB39276, PAB30712, PAB44700, PAB40077, PAB45936, PAB41857, PAB36269, Bioswamp); p-Smad2 antibody (ab188334, Abcam); p-Smad3 antibody (9520T, CST); Goat anti-Rabbit immunoglobulin G (SAB43714, bioswamp); MaxVision TM secondary antibody and horseradish peroxidase (HRP)-Polymer (Kit-5020, Maixin company).

The equipment used included: Electronic balance (JM-A3002, Zhuji Company); blood glucose monitor; surgical straight scissors, tissue forceps (J21070, J41050, Admiralty); inverted fluorescence microscope (DMILLED, Leica company); high-speed refrigerated centrifuge, microplate reader, plate washer (Icen-24R, AMR-100, APW-200, Hangzhou Aosheng Company); constant temperature oven (DHG-9023A, Shanghai Yiheng Company); automatic tissue dehydrator, stall Slice baking machine (TKD-TSF, TKD-TK, Hubei Kangqiang Company); paraffin slicer (RM2235, Leica Microsystems); biological tissue embedding machine-freezer (TB-718L, Thailand Technology); automatic chemiluminescence analyzer (HT8300/8500; Hongji Company); water bath (H.SWX-600BS, Shengke Company); and ultrapure water device (ULUPURE, MilliPORE France).

### Construction and Identification of Rat Diabetes Model

The study included 36 rats, half male and half female, 8 weeks old, and weighing about 250 g each. The animals were obtained from Three Gorges University with laboratory animal license number: SYXK (E) 2018-0104, certificate No. 42010200003097. Rats were raised under SPF conditions. The housing conditions were: temperature of 22–26°C, relative humidity of 50–60%, artificial light and dark conditions for 12 h, and adaptive feeding for 1 week. 6 rats were selected as the normal control group, and the other 30 rats were the model group. They were given high-sugar and high-fat diet (10% sucrose, 10% lard, 10% egg yolk powder, 1.5% cholesterol 0.5% bile Sodium, 68% full feed) for 4 weeks (8). After 4 weeks, the model group was starved for more than 12 h, but with drinking, and then intraperitoneally injected with 35 mg/kg freshly prepared 1% STZ solution (6). After 72 h, rats were successfully modeled for diabetes with random blood glucose ≥16.7 mmol/L from the posterior tail vein.

### Drug Intervention and Sample Collection

After the model was successfully generated, the rats were divided into 6 groups for drug intervention, each with 6 rats: ① Normal control group (NC): fed with ordinary feed; ② Diabetes model group (DM): equal volume of drinking water gavage. Gavaged once a day for 8 consecutive weeks and administered high-fat diet; ③ JTTMP low-dose group (DM+JTTMP 63 mg/kg): JTTMP 63 mg/mL suspension 1 ml/(kg·d) was given by intragastric administration for 8 weeks, and high-fat diet was administered; ④JTTMP middle-dose group (DM+JTTMP 126 mg/kg): JTTMP 126 mg/mL suspension 1 ml/(kg·d) was intra-gastrically administered for 8 weeks and the high-fat diet was continued; ⑤ JTTMP high-dose group (DM+JTTMP 252 mg/kg): JTTMP 252 mg/mL suspension 1 mL/(kg·d) was gavaged for 8 weeks, and high-fat diet was administered; and ⑥ Metformin group (DM+Met): 54.3 mg/ml Metformin aqueous solution of 1 ml/(kg·d) gavaged and administered for 8 weeks, and high-fat diet was administered.

After the drug treatments were completed, the rats in each group were sacrificed, and the serum was frozen for later use. The left lung tissue was fixed with 4% paraformaldehyde, and the right was stored at −80°C until analysis.

### General Behavior Observation

During the experiment, the activity, eating, drinking, and excretion of the rats were observed, and the weight of the rats was recorded every day. The comparison of weight changes in each group was statistically analyzed.

### Fasting Blood Glucose Test in Rats

Blood from the tail vein was collected in each group of rats before and after drug intervention, the fasting blood glucose was measured by the test strip method, and the blood glucose value data was recorded.

### The Detection of Inflammatory Factors IL-6, TNF-α, IL-1β in Serum and Lung Homogenate of Each Group of Rats by ELISA

The serum and lung tissue homogenate of rats from each group was collected, and ELISA was performed using 50 μL of each standard and sample solution, and phosphate-buffered saline as a negative control. An equal volume of enzyme-labeled IL-6 or TNF-α and IL-1β antibodies was added to the relevant sample and standard wells. Plates were sealed with film and incubated at 37°C for 30 min. Plates were spun and washed. The developer (50 μL) was added and incubated at 37°C for 10 min. Finally, 50 μL of stop solution was added and the absorbance (OD value) at a wavelength of 450 nm was measured. A standard curve was used to calculate the concentrations of IL-6, TNF-α, and IL-1β in the sample.

### Observation of Morphological Changes of Rat Lung Tissue by Hematoxylin and Eosin Staining

The lung tissue of rats from each group was collected, fixed with 4% paraformaldehyde, and embedded in paraffin. As per routine practice, tissue sections were deparaffinized, stained with hematoxylin for 3–6 min, washed for 1–2 min, differentiated with 1% hydrochloric acid alcohol for 1–3 s, promoting liquid returned to blue for 5–10 s, washed with running water for 15–30 s, stained with 0.5% eosin solution for 2–3 min, washed with distilled water for 1–2 s, then 80% ethanol for 15–30 s, then 95% ethanol for 15–30 s, and finally anhydrous ethanol for 1–2 s. After drying, sections were sealed with neutral gum. The pathology of lung tissues such as the alveolar wall and inflammatory infiltration were analyzed under a microscope.

### Western Blot Detection of TGF-β1, SnoN, Smad2, Smad3, Smad7, p-Smad2, p-Smad3, p-Smad7 Signal Protein in Rat Lung Tissue

Rat lung tissues were collected from each group, briefly homogenized, and protein extraction reagent at 5 μl/mg and protease inhibitor phenylmethylsulfonyl fluoride was added at a final concentration of 1 mM. Tissues were lysed for 15 min on ice and centrifuged at 4°C, 13800 r/min for 15 min. The protein concentration of the supernatant was quantified and adjusted. Protein samples had loading buffer added and were denatured at 100°C for 5 min. Electrophoresis separation was performed by 120 g/L sodium dodecyl sulfate-polyacrylamide gel electrophoresis, the nitrocellulose membrane was transferred, and 5% skimmed milk powder was used to block for 2 h at room temperature, then TGF-β1 antibody (1:1000), Smad2 antibody (1:1000), Smad3 antibody (1:1000), Smad7 antibody (1:1000), p-Smad2 antibody (1:1000), p-Smad3 antibody (1:1000), p-Smad7 antibody (1:1000), and GAPDH antibody (1:1000) were incubated overnight on samples at 4°C. The membrane was washed with tris-buffered saline with 0.1% Tween^®^ 20 Detergent (TBST) thrice and incubated with HRP-labeled secondary antibody (1:20000) for 2 h at room temperature. The membrane was again washed with TBST thrice. Finally, the enhanced chemiluminescence was analyzed after exposure in the darkroom.

### Statistical Analysis

All data were analyzed by SPSS 21.0. The *t*-test for the comparison of two sample means was performed. Data were expressed as the $\bar{\text{x}}\pm \text{s}$ , the comparison between multiple groups was determined by one-way analysis of variance, and *P*<0.05 indicates that the difference was statistically significant.

## Results

### Changes in Body Weight of Rats in Each Group Over Time

After the generation of the model, the bodyweight of the DM group gradually decreased over time. After 4 weeks, the body weight in the DM group was significantly lower than that of the NC group at each time point (*P*<0.01). Compared with the DM group, the extent of weight loss in the DM+JTTMP 126 mg/kg group was reduced (*P*<0.05), and the weight loss in the DM+JTTMP 252 mg/kg and the DM+Met groups were significantly improved. In summary, the weight of the DM+Met group at each time point was much higher than that of the DM group (*P*<0.05, **Table 1**).

**Table 1:** The effect of JTTMP on the body weight of diabetic rats induced by STZ ( $\bar{\text{x}}\pm \text{s}$ , n=6).

Group	BW (g)				
	Before drug intervention	2 week	4 week	6 week	8 week
NC	490.18 ± 12.06	537.84 ± 7.53	563.32 ± 7.38	575.74 ± 12.91	580.48 ± 13.31
DM	560.58 ± 2.41	422.92 ± 10.16	342.76 ± 3.40^**^	274.38 ± 3.00^**^	216.32 ± 5.32^**^
DM+JTTMP 63 mg/.kg^-1^	552.62 ± 6.28	415.32 ± 7.73	340.58 ± 4.06	285.44 ± 6.09	236.84 ± 5.58
DM+JTTMP 126 mg/.kg-1	555.40 ± 3.56	418.62 ± 2.86	366.68 ± 1.59^#^	316.62 ± 2.91^#^	271.2 ± 3.19^#^
DM+JTTMP 252 mg/.kg-1	552.98 ± 8.81	419.40 ± 4.46	380.70 ± 3.27^#^	344.72 ± 3.60^##^	309.42 ± 5.26^##^
DM+Met	552.48 ± 8.84	424.76 ± 13.04	386.48 ± 6.60^#^	361.98 ± 9.04^##^	321.36 ± 8.51^###^

vs NC group, **P<0.01; vs DM group, ^#^P<0.05, ^##^P<0.01, ^###^P<0.001.

### Effect of JTTMP on Blood Sugar of Diabetic Rats

After the model was successfully established, it was found that the glucose level of the DM group (20.15 ± 2.48 mmol/L) was higher than that of the NC group (5.68 ± 0.68 mmol/L, *P*<0.001). The glucose level in the DM group remained higher than in the NC group after 8 weeks (*P*<0.001). There was no obvious difference in blood glucose between the DM group and the treated group before the drug intervention; however, for 8 weeks after the drug intervention, the glucose in the DM+JTTMP 126 mg/kg group decreased to be lower than the DM group (*P*<0.05). The blood glucose level in the DM+JTTMP 252 mg/kg and DM+Met groups further decreased to be significantly lower than that of the DM group (*P*<0.01, **Table 2**).

**Table 2:** Comparison of FPG among the different groups ( $\bar{\text{x}}\pm \text{s}$ , n=6).

Group	FDP (mmol/L)	
	Before drug intervention	8 week
NC	5.68 ± 0.68	5.65 ± 0.89
DM	20.15 ± 2.48^***^	19.38 ± 1.37^***^
DM+JTTMP 63 mg/.kg^-1^	18.44 ± 1.29	17.02 ± 1.14
DM+JTTMP 126 mg/.kg-1	18.48 ± 1.35	14.95 ± 1.55^#^
DM+JTTMP 252 mg/.kg-1	20.74 ± 3.29	13.94 ± 0.96^##^
DM+Met	19.26 ± 2.23	11.76 ± 1.01^##^

vs NC group, ***P<0.001;vs DM group, ^#^P<0.05, ^##^P<0.01.

### Expression of Inflammatory Factors IL-6, TNF-α, IL-1β in Serum and Lung Homogenate of Rats

**Figure 1:**
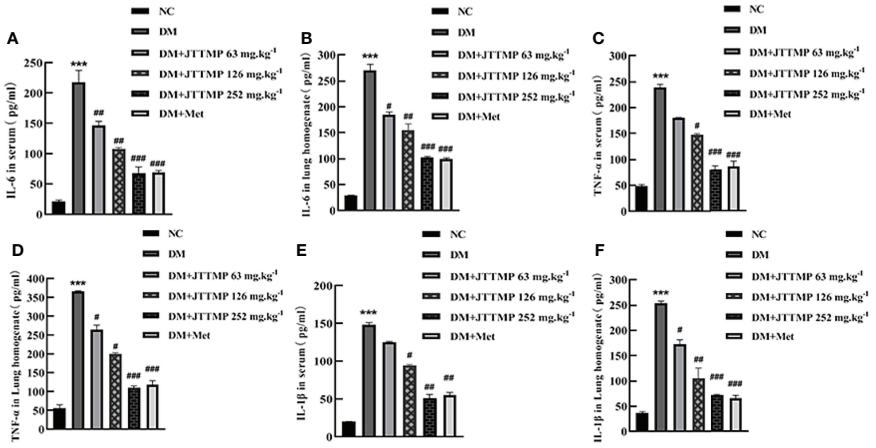
Expression of inflammatory factors IL-6, TNF-α, and IL-1β in serum and lung homogenate of rats from different groups. **(A, B)** The concentration of IL-6 in serum and lung homogenate of rats from different groups. **(C, D)** The concentration of TNF-α in serum and lung homogenate of rats from different groups. **(E, F)** The concentration of IL-1β in serum and lung homogenate of rats from different groups. *vs* NC group, **P<0.001; *vs* DM group, ^#^ *P<*0.05, ^##^ *P<*0.01.

To clarify whether JTTMP plays a role in improving resistance to inflammatory injury in diabetes, we first detected the level of IL-6, TNF-α, and IL-1β in the serum and lung tissue in each group of rats by ELISA. The results showed that the expressions of IL-6, TNF-α, and IL-1β in serum and lung homogenate in the DM group were higher than those of the NC group (*P*<0.001). The level of inflammatory factors in the DM+JTTMP and DM+Met groups was significantly lower than those in the DM group (*P*<0.05), and as the dose of JTTMP increased, the expression of inflammatory factors gradually decreased. In the high-dose group with JTTMP 252 mg/kg, inflammatory factors were significantly reduced (*P*<0.001) to similar levels close to the normal level (**Figure 1**).

### Changes in Lung Tissue Structure in Each Group of Diabetic Rats

**Figure 2:**
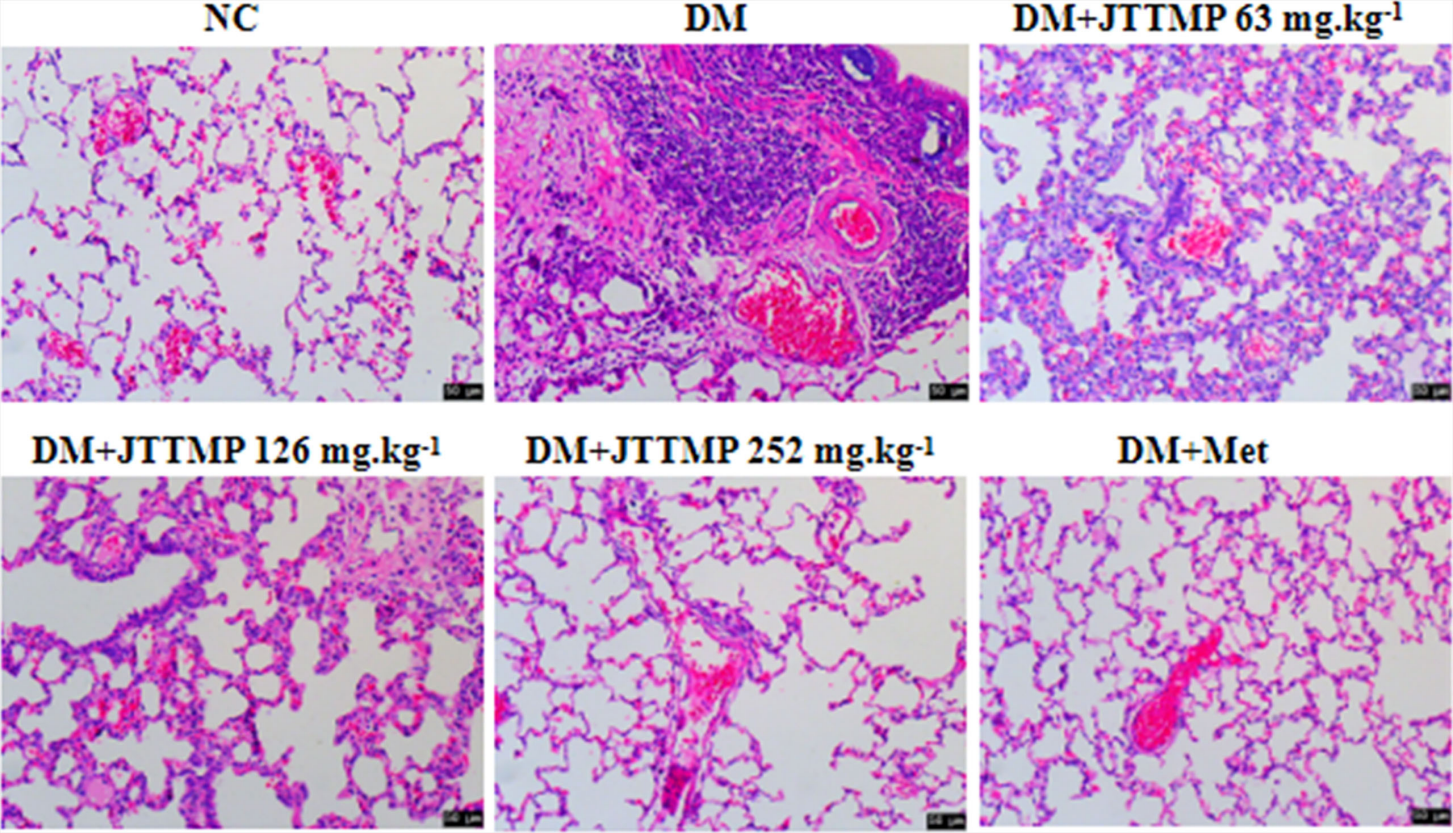
Microphotographs showing structural changes with H&E staining in lung tissue of rats from different groups (200×).

To further define the role of JTTMP in the process of diabetic inflammatory injury, changes to the lung structure of rats were observed by H&E staining. The lung tissue in the NC group showed no obvious abnormality. In comparison with the NC group, the alveolar cavities in the DM group were reduced, the alveolar compartments were thickened, and some of the alveolar cavities were atrophied or collapsed with a large quantity of infiltrated inflammatory cells. In comparison with the DM group, lung tissue lesions in the DM+JTTMP and DM+Met groups were improved. As the dose of JTTMP increased, lung tissue lesions were significantly improved, inflammatory cell infiltration decreased, and alveolar spacing returned to normal (**Figure 2**).

### JTTMP Reduced Diabetic Lung Injury Through SnoN Protein and Downstream TGF-β1/Smads Signaling Pathway

**Figure 3:**
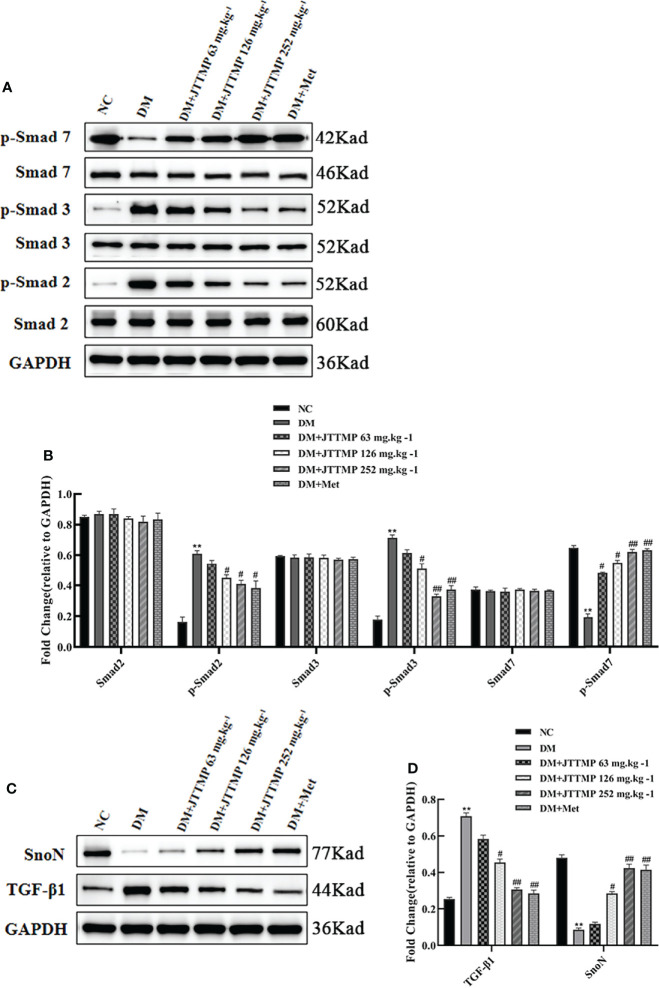
Western blot analysis of TGF-β1/Smads expression in lung tissue of rats from different groups. **(A)** Smads protein family expression in the lung of rats from different groups; **(B)** Quantitative analysis of Smads protein family expression; **(C)** TGF-b1 and SnoN expression in lung tissue of rats from different groups; **(D)** Quantitative analysis of TGF-b1 and SnoN expression. **P<0.01; *vs* DM group, ^#^ *P<*0.05, ^##^ *P<*0.01.

To explore the molecular mechanism of JTTMP in reducing diabetes lung injury, the key proteins in the TGF-β1/Smads pathway were assessed by Western blot. There were no significant changes in the expression of Smad2, Smad3, and Smad7 in each group, while phosphorylated Smad2 and Smad3 expressions were much higher in the DM group than in the NC group (*P*<0.01, **Figures 3A, B**). After treatment, the levels of p-Smad2 and p-Smad3 in the DM+JTTMP and DM+Met groups were much lower than in the DM group, and their expression level gradually decreased with the increase of the dose of JTTMP (*P*<0.05, **Figures 3A, B**). The level of p-Smad7 in the DM group was much lower, and p-Smad7 in the DM+JTTMP and DM+Met groups increased after treatment and also showed a dose-dependent effect of JTTMP (*P*<0.05, **Figures 3A, B**). To further clarify the targets of Smad protein interaction, we assessed the expression of SnoN and TGF-β1. The expression of SnoN in the DM group was significantly lower than in the NC group (*P*<0.01) but was increased in the DM+JTTMP and DM+Met groups after treatment (*P*<0.05, **Figures 3C, D**), which was consistent with the expression of p-Smad7. The expression of TGF-β was significantly increased in the DM group but decreased with the increase of JTTMP (*P*<0.05, **Figures 3C, D**).

## Discussion

Diabetes is very often a chronic disease. With the improvement of people’s living standards, the incidence of diabetes is gradually increasing. According to statistics, in China, the incidence of diabetes has reached 2%, there are 40 million diagnosed diabetic patients, and this is increasing at a rate of 1 million per year. As it is predicted that by 2025, the number of diabetic patients in the world will reach 370 million, diabetes has become a serious threat to global public health (9, 10).

This study shows that an STZ-induced rat model of diabetes had a clear decrease in body weight and increase in blood glucose, while after 8 weeks of JTTMP drug intervention blood glucose level was significantly decreased, and the extent of weight loss was reduced. These results are consistent with those described in the literature (11).

Recent studies have shown that the lung is also damaged by diabetes (12, 13). Diabetic lung tissue has fibrotic changes similar to diabetic cardiomyopathy (14). Our results found that the alveolar cavity of the lung tissue of diabetic rats was reduced, inflammatory cells had infiltrated, the alveolar compartment was thickened, and a large number of collagen fibers had proliferated, showing pulmonary fibrosis. These results are consistent with previously reported results of diabetic lung damage (12–14). At the same time, we measured the levels of IL-6, TNF-α, and IL-1β in the serum and lung homogenate of diabetic rats. With the increase of the dose of JTTMP, the expression of inflammatory factors gradually decreased. Therefore, JTTMP was shown to alleviate diabetic lung injury and improve the prognosis of diabetic lung injury.

Smad, the downstream signaling molecule of the TGF-β family, is the sole substrate of TGF-β. Under the stimulation of a variety of related factors, TGF-β firstly binds to TGF-β receptor II on the cell membrane and activates the TGF-β receptor, directly phosphorylates Smad2 and Smad3, finally forms a trimer with Smad4 and transfers to the nucleus, thereby internally regulating the transcription of the corresponding target gene. Smad7 is an inhibitory Smad protein, which can reduce the expression of TGF-β through negative feedback regulation of the TGF-β signal transduction pathway, thereby playing a protective effect on diabetic lung tissue.

Studies have shown that there is a higher level of TGF-β1 in diabetic patients than normal. Among the related cytokine networks, TGF-β1 is recognized as the most closely related factor that mediates the formation, occurrence, and development of fibrosis (15). TGF-β1 can stimulate fibroblasts to divide and proliferate, promote their activation into myofibroblasts, and reduce their degradation. The Smads protein family is currently the only known type I receptor related to TGF-β1 that can mediate its signal from the cell membrane receptor to the corresponding cell nucleus (16, 17). Studies have shown that the Smads proteins family contains 9 members, and TGF-β1 can activate Smad2/3 and promote fibrosis. In addition, the main inhibitory regulatory protein Smad7 and the TGF-β1/Smads signaling pathway coordinate and cooperate to perform signal transduction (18–20). Our study shows that the level of TGF-β protein in the DM group was higher than that of the NC group, indicating that diabetic lung injury may be associated with the increase of TGF-β. The expression of Smad2/3 in the diabetic rats increased, while the expression of Smad7 decreased, suggesting that TGF-β participates in the formation of diabetic lung fibrosis by upregulating Smad2/3 and downregulating Smad7 expression. Following JTTMP treatment, TGF-β can reduce the level of Smad2/3 and increase the level of Smad7. At the same time, blood sugar levels are reduced, and the effect may be more significant as treatment time is extended. These results support the theoretical explanation of the TGF-β1/Smads pathway in the previously mentioned literature (18–20).

The transcriptional co-repressor SnoN protein belongs to the Ski protein family. Its most important function is to negatively regulate TGF-β signal transduction by binding to the Smad protein (21). Ski and SnoN simultaneously interact with R-Smad (Smad2/3) through their N-terminal region and with co-Smad (Smad4) through a SAND-like domain (22). In this way, it prevents the function of the Smad complex and activates TGF-β target genes. In addition, the binding of SnoN can also stabilize the inactive Smad heteromers, to prevent the further binding of the active Smad complex (23). In this study, when diabetic lung injury occurred, the TGF-β1/Smads signaling pathway interacted with SnoN protein, thereby inhibiting the inflammatory signal and reducing the further occurrence of lung injury.

Diabetes is categorized as a “wasting-thirst” disorder in Traditional Chinese Medicine. The pathogenesis of diabetes in Traditional Chinese Medicine mainly includes yin deficiency and fire prosperity, and lung and kidney yin deficiency. The JTTMP treatment is composed of three Chinese medicines including Huanglian, Chuanqiong, and Ligustrum lucidum. The combination of various drugs has the effect of nourishing yin and removing blood stasis (24). JTTMP can effectively treat diabetes, but whether it is beneficial in the treatment of diabetes with lung injury still needs to be explored. This study used STZ induction to establish a rat diabetic model, and following intervention with JTTMP it was found to be effective at alleviating diabetic lung damage through SnoN protein and downstream TGF-β1/Smads pathway activity. Further research on the TGF-β/Smads signaling pathway will help clarify the mechanism of diabetic lung injury and provide new targets for the prevention and treatment of diabetic lung injury.

## Data Availability Statement

The raw data supporting the conclusions of this article will be made available by the authors, without undue reservation.

## Ethics Statement

The animal study was reviewed and approved by Hubei University of Chinese Medicine.All animal experimentation was conducted in accordance with accepted standards of humane animal care, as outlined in the Ethical Guidelines.

## Author Contributions

ND: Study design, contributed to statistical analysis, and manuscript drafting.; CZ: Contributed to data analysis and helped in results, discussion, and drafting. All authors critically reviewed and agreed on the final version of the manuscript.

## Funding

This work was funded by Hubei Natural Science Foundation key project (2019 CFB633), Wuhan Health and Family Planning Commission Key Scientific Research Project (WZ20A06), and Wuhan Young and middle-aged Medical Talents Training Project ([2013]35).

## Conflict of Interest

The authors declare that the research was conducted in the absence of any commercial or financial relationships that could be construed as a potential conflict of interest.

## Publisher’s Note

All claims expressed in this article are solely those of the authors and do not necessarily represent those of their affiliated organizations, or those of the publisher, the editors and the reviewers. Any product that may be evaluated in this article, or claim that may be made by its manufacturer, is not guaranteed or endorsed by the publisher.
